# Deep learning-based quantification of epicardial adipose tissue volume from non-contrast computed tomography images: a multi-centre study

**DOI:** 10.1093/ehjdh/ztaf116

**Published:** 2025-10-13

**Authors:** Shuang Leng, Nicholas Cheng, Eddy Tan, Lohendran Baskaran, Lynette Teo, Min Sen Yew, Kee Yuan Ngiam, Weimin Huang, Ping Chai, Ching Ching Ong, Ching Hui Sia, Malay Singh, Yan Ting Loong, Nur A S Raffiee, Xiaomeng Wang, John Allen, Swee Yaw Tan, Mark Chan, Hwee Kuan Lee, Liang Zhong

**Affiliations:** CVS.AI, National Heart Research Institute Singapore, National Heart Centre Singapore, 5 Hospital Drive, Singapore 169609, Singapore; Duke-NUS Medical School, 8 College Road, Singapore 169857, Singapore; Bioinformatics Institute, Agency for Science, Technology and Research (A*STAR), 30 Biopolis Street, Matrix, Singapore 138671, Singapore; Bioinformatics Institute, Agency for Science, Technology and Research (A*STAR), 30 Biopolis Street, Matrix, Singapore 138671, Singapore; CVS.AI, National Heart Research Institute Singapore, National Heart Centre Singapore, 5 Hospital Drive, Singapore 169609, Singapore; Duke-NUS Medical School, 8 College Road, Singapore 169857, Singapore; Department of Cardiology, National Heart Centre Singapore, 5 Hospital Drive, Singapore 169609, Singapore; Department of Diagnostic Imaging, National University Hospital, 5 Lower Kent Ridge Road, Singapore 119074, Singapore; Yong Loo Lin School of Medicine, National University of Singapore, 10 Medical Drive, Singapore 117597, Singapore; Department of Cardiology, Tan Tock Seng Hospital, 11 Jalan Tan Tock Seng, Singapore 308433, Singapore; Yong Loo Lin School of Medicine, National University of Singapore, 10 Medical Drive, Singapore 117597, Singapore; Department of Surgery, National University Hospital, 5 Lower Kent Ridge Road, Singapore 119074, Singapore; Institute for Infocomm Research, Agency for Science, Technology and Research (A*STAR), 1 Fusionopolis Way, Connexis South Tower, Singapore 138632, Singapore; Yong Loo Lin School of Medicine, National University of Singapore, 10 Medical Drive, Singapore 117597, Singapore; Department of Cardiology, National University Heart Centre, 5 Lower Kent Ridge Road, Singapore 119074, Singapore; Department of Diagnostic Imaging, National University Hospital, 5 Lower Kent Ridge Road, Singapore 119074, Singapore; Yong Loo Lin School of Medicine, National University of Singapore, 10 Medical Drive, Singapore 117597, Singapore; Yong Loo Lin School of Medicine, National University of Singapore, 10 Medical Drive, Singapore 117597, Singapore; Department of Cardiology, National University Heart Centre, 5 Lower Kent Ridge Road, Singapore 119074, Singapore; Bioinformatics Institute, Agency for Science, Technology and Research (A*STAR), 30 Biopolis Street, Matrix, Singapore 138671, Singapore; CVS.AI, National Heart Research Institute Singapore, National Heart Centre Singapore, 5 Hospital Drive, Singapore 169609, Singapore; CVS.AI, National Heart Research Institute Singapore, National Heart Centre Singapore, 5 Hospital Drive, Singapore 169609, Singapore; CVS.AI, National Heart Research Institute Singapore, National Heart Centre Singapore, 5 Hospital Drive, Singapore 169609, Singapore; Duke-NUS Medical School, 8 College Road, Singapore 169857, Singapore; Duke-NUS Medical School, 8 College Road, Singapore 169857, Singapore; Duke-NUS Medical School, 8 College Road, Singapore 169857, Singapore; Department of Cardiology, National Heart Centre Singapore, 5 Hospital Drive, Singapore 169609, Singapore; Yong Loo Lin School of Medicine, National University of Singapore, 10 Medical Drive, Singapore 117597, Singapore; Department of Cardiology, National University Heart Centre, 5 Lower Kent Ridge Road, Singapore 119074, Singapore; Bioinformatics Institute, Agency for Science, Technology and Research (A*STAR), 30 Biopolis Street, Matrix, Singapore 138671, Singapore; School of Computing, National University of Singapore, 13 Computing Drive, Singapore 117417, Singapore; International Research Laboratory on Artificial Intelligence, Agency for Science, Technology and Research (A*STAR), 1 Fusionopolis Way, Connexis South Tower, Singapore 138632, Singapore; Centre for Frontier AI Research, Agency for Science, Technology and Research (A*STAR), 1 Fusionopolis Way, Connexis North Tower, Singapore 138632, Singapore; School of Biological Sciences, Nanyang Technological University, 60 Nanyang Drive, Singapore 637551, Singapore; CVS.AI, National Heart Research Institute Singapore, National Heart Centre Singapore, 5 Hospital Drive, Singapore 169609, Singapore; Duke-NUS Medical School, 8 College Road, Singapore 169857, Singapore; Department of Biomedical Engineering, National University of Singapore, 4 Engineering Drive 3, Singapore 117583, Singapore

**Keywords:** Epicardial adipose tissue, Deep learning, Non-contrast CT, Coronary artery disease

## Abstract

**Aims:**

Epicardial adipose tissue (EAT), located within the pericardial sac, has emerged as a biomarker for coronary artery disease (CAD) progression. This study aimed to develop and validate a deep learning-based system for automated EAT volume quantification using non-contrast computed tomography (NCCT) scans from a large, multi-centre, pan-Asian cohort.

**Methods and results:**

A total of 1243 NCCT patient scans from three centres were used to train and internally validate a deep learning model based on 3D UNet++ architecture for pericardium segmentation, followed by intensity thresholding to derive EAT volume. Epicardial adipose tissue quantification required ∼30 s per scan. The final model was evaluated on an external testing cohort of 160 patients, including 90 non-Asian individuals. In this cohort, AI-predicted EAT volumes showed excellent agreement with expert annotations (*r* = 0.975; *P* < 0.0001). The Bland–Altman analysis demonstrated a mean bias of −5.2 cm^3^with 95% limits of agreement from −25.1 to 14.7 cm^3^. Among the non-Asian subgroup, model performance remained strong (*r* = 0.970; bias, −3.2 cm^3^; limits of agreement, −25.1–18.7 cm^3^). AI-derived EAT volume was independently associated with obstructive CAD (odds ratio 1.11; 95% confidence interval, 1.04–1.19; *P* = 0.004), after adjusting for confounders. The global χ^2^ statistic increased from 81.7 with coronary calcium score alone to 93.3 when EAT volume was added (*P* = 0.001), indicating improved risk prediction.

**Conclusion:**

We developed and validated a deep learning system for automated EAT volume quantification from NCCT scans. The model demonstrated high accuracy and generalizability across ethnically diverse populations, supporting its potential for routine EAT assessment and CAD risk stratification.

**Trial Registration:**

ClinicalTrials.gov Identifier: NCT05509010.

## Graphical Abstract

**Graphical Abstract:**
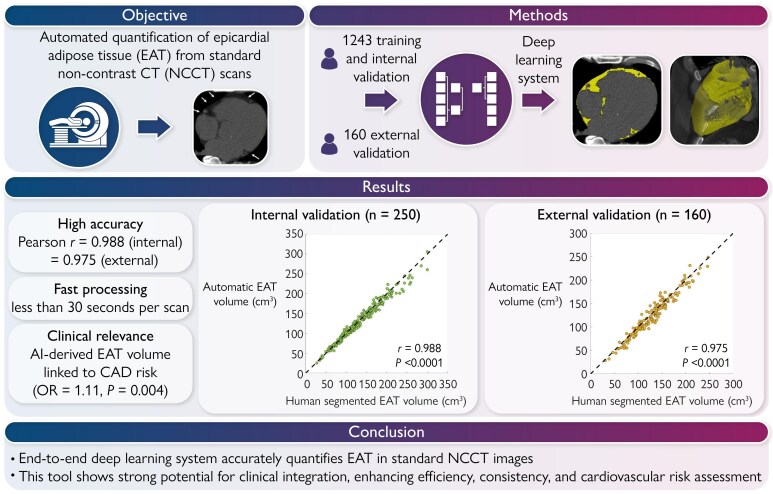
Deep learning-based quantification of epicardial adipose tissue volume from non-contrast computed tomography images: a multi-centre study.

## Introduction

Epicardial adipose tissue (EAT) is a visceral fat deposit distributed within the pericardium of the heart. Increased EAT volume has been shown to be significantly related to major adverse cardiovascular events (MACEs),^1^ particularly in patients with coronary artery disease (CAD).^2^ A prior meta-analysis using computed tomography (CT) images with >41 000 participants over 70 studies showed an association between EAT volume and coronary artery stenosis, myocardial ischaemia, and MACE.^3^ Patients with any CAD manifestation had a higher mean volume of EAT than those without CAD-related outcomes. Another study found that in patients with suspected CAD, EAT volume was significantly and positively associated with the risk of obstructive CAD, independent of traditional risk factors and coronary artery calcium.^4^ Additionally, there are reports suggesting that EAT may represent a new therapeutic target in cardiovascular diseases.^5,6^

Despite the clinical significance of EAT, its quantification remains a manual and labour-intensive endeavour, requiring expertise and time. Consequently, the integration of EAT measurement into clinical practice has been hindered by the absence of reliable, time-efficient, and fully automated quantification methodologies.^7^ Although semi-automated approaches have been extensively explored,^8,9^ there still lacks a robust, expedited, and rigorously validated solution.^10^

Deep learning-based methods have been applied to quantify EAT volume,^10,11^ but several challenges still remain. One of the most significant is accurate segmentation of the apical (inferior) region, which often presents with poor contrast and indistinct anatomical boundaries, making it difficult to distinguish from surrounding tissues. We recognize there are no prior studies that have specifically quantified the quality of apex segmentation in the context of EAT volume assessment. Additionally, most studies on EAT quantification have focused on White or European populations, despite known ethnic differences in fat distribution and cardiovascular risk.^12^ Given that Asians may exhibit greater ectopic fat accumulation and cardiometabolic risk at lower body mass index (BMI),^13^ population-specific evaluation is essential to ensure accurate deep learning-based EAT quantification and clinical risk stratification.

Our study aimed to (i) develop a deep learning-based solution for the automated quantification of EAT volume from non-contrast CT (NCCT) scans with improved segmentation accuracy in large Asian populations and (ii) evaluate its performance and clinical utility across diverse populations. We hypothesized that AI-derived EAT volume is significantly predictive of obstructive CAD. Developed and rigorously validated across diverse patient cohorts spanning multiple countries and centres, our approach has the potential to facilitate seamless integration into routine clinical practice.

## Methods

### Study population

Our study population included 1243 patients (mean age, 58 ± 11 years; 813 male) enrolled in the APOLLO trial (ClinicalTrials.gov: NCT05509010), a hybrid retrospective-prospective, open-label, observational, multi-centre study conducted across Singapore’s three largest cardiac institutions.^14^ The trial was designed to develop a national AI-driven platform for coronary CT angiography (CCTA) applications in a multi-ethnic Asian population. Patients with suspected or known CAD were enrolled, and imaging was performed as part of routine clinical care. Exclusion criteria in retrospective patient recruitment included acute coronary syndrome, BMI exceeding 40 kg/m^2^, or a history of percutaneous or surgical intervention for CAD. In prospective patient recruitment, subjects were excluded if they had complex congenital heart disease, planned invasive angiography for reasons other than CAD, non-cardiac illness with a life expectancy of less than 2 years, or a cardiac event and/or coronary revascularization and/or valvular repair/replacement prior to CT scan. Additional exclusion criteria were current pregnancy, glomerular filtration rate of ≤30 mL/min, known allergies to iodinated contrast agents, or contraindications to beta blockers, nitroglycerin, or adenosine.

To minimize training bias and ensure balanced representation across the EAT volume spectrum, we stratified the dataset into two groups based on a threshold of 125 cm^3^, which represents the average EAT volume in the overall cohort. Group 1 included patients with EAT volume of ≥125 cm^3^, and Group 2 included those with EAT volume of <125 cm^3^. Patients from each group were then randomly assigned to the training and internal testing sets using an 80:20 split. This stratified randomization ensured similar EAT volume distributions between the training and internal testing sets. Of the 1243 patients, 993 were allocated to the training set, and the remaining 250 were used to test model performance. The study protocol complied with the Declaration of Helsinki and was approved by the SingHealth Centralised Institutional Review Board. Informed consent was obtained from each prospectively recruited participant, or a waiver of consent was granted for the use of de-identified retrospective data.

Following AI model development, external validation was performed using 160 previously unseen patient scans, comprising (i) 115 patients (mean age, 60 ± 9 years; 104 male) from an ongoing prospective trial where post-acute myocardial infarction patients were recruited across eight centres in two countries (Singapore and New Zealand) between July 2021 and March 2023;^15,16^ (ii) 25 patients from the University of Porto (SAFE-CT study, ClinicalTrials.gov: NCT06438393); and (iii) 20 patients from Brazil, sourced from the Cardiac Fat Database—Computed Tomography.^17^ Among these 160 cases, 90 were from non-Asian individuals.

**Figure 1:**
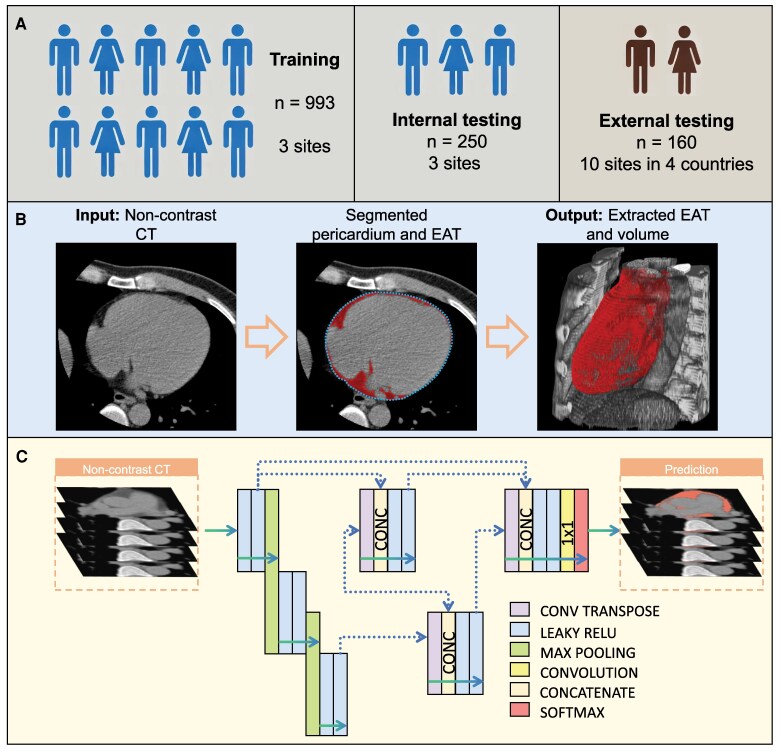
(*A*) Overview of the training, internal testing, and external testing datasets. (*B*) Overall workflow for epicardial adipose tissue prediction. (*C*) Network architecture used in the current study. EAT, epicardial adipose tissue.

A graphic summarizing model development, testing, and external validation is presented in *Figure 1*.

### Computed tomography image acquisition and annotation

All patients enrolled in the APOLLO study underwent both NCCT and contrast-enhanced CCTA during the same scanning session. The imaging protocol adhered to the Society of Cardiovascular Computed Tomography (SCCT) guidelines,^18^ and scans were acquired using either a Toshiba Aquilion One scanner, a Siemens Somatom Force scanner, or a Siemens SOMATOM Definition Flash scanner with prospective ECG triggering. Raw image data were reconstructed with a slice thickness of 3.0 mm. The specific CT scanners and acquisition parameters are detailed in Supplementary material online, *Table S1*. Obstructive CAD was defined as ≥50% luminal narrowing in any of the major coronary arteries.

The pericardium was manually segmented in all NCCT scans used for training and testing. All annotations were performed by three certified analysts from the CardioVascular Systems Imaging and Artificial Intelligence (CVS.AI) research core lab. Each analyst has over 3 years of experience in cardiac CT imaging and is SCCT Level 1 certified. All segmentations were subsequently reviewed by three cardiologists or radiologists, each with more than 15 years of experience in cardiac CT interpretation.

Manual pericardium segmentation was performed using the software 3D Slicer (version 4.11.20210226) (see Supplementary material online, *Figure S1*). First, the NCCT image window settings were adjusted to a width of 350 Hounsfield units (HU) and a level of 40 HU to improve visualization of the pericardium.^11^ Second, the superior and inferior limits of the heart (pulmonary artery bifurcation and interventricular groove, respectively) were defined, determining the region of interest for pericardium segmentation within the NCCT scan. Third, the pericardium was manually delineated on every 5th axial slice. In the inferior regions of the heart where the pericardium is more difficult to distinguish,^11^ additional annotations were performed on consecutive slices to improve segmentation accuracy. The sagittal view was also used alongside the axial view to ensure precise delineation (see Supplementary material online, *Figure S1*). Following this, the full segmentation mask was obtained for every axial slice by smoothly interpolating between the manually annotated slices. Next, the EAT mask was obtained within the pericardium mask by applying a threshold range of −190 to −30 HU, which is the established range for adipose tissues on scans acquired at 120 kV.^19^ Finally, the resulting EAT volume was calculated by summing the EAT voxels and multiplying by the dimensions of each voxel.

### Data preprocessing

#### Resampling

Preprocessing was performed to standardize the input CT images and corresponding pericardium masks for efficient training of the deep learning model. The first step involved isotropic resampling to achieve uniform voxel spacing of 1.6 × 1.6 × 1.6mm. Trilinear interpolation was applied to the CT images, while nearest neighbour interpolation was used for the binary pericardium masks to preserve label integrity. As a result of resampling, the image volumes varied in shape. To ensure consistent input dimensions, both CT images and pericardium masks were subsequently padded and/or cropped along all three axes to obtain a final size of 128 × 128 × 128 voxels. To enhance model robustness, the intensity values of the CT images were clipped to the range of [−800, 1200] HU, which encompasses the expected range of soft tissue, fat, and calcified structures while reducing the influence of extreme outliers and noise. The clipped values were then linearly normalized to the range [0, 1] on a per-voxel basis to facilitate stable training. This normalization step was applied only to the CT images and not to the binary masks.

#### Redundant class

Initial testing revealed that the AI-predicted pericardium segmentation was less accurate in the inferior (apical) region compared with the middle and superior regions. Additionally, the 15–20% of EAT slices located at the most inferior region account for more than 20% of the total EAT volume (see Supplementary material online, *Figure S2*), underscoring the clinical and methodological importance of accurate segmentation in this region. To improve model learning in this challenging area, we introduced a redundant class label based on HU thresholding within the range of −198 to −80 HU, applied specifically to the inferior 20% of axial slices in each scan (see Supplementary material online, *Figure S3*). This redundant class was defined as a separate category from the background, enabling the model to better distinguish the pericardium boundary in this region. A similar approach has been adopted in previous work, where a redundant class was used to improve deep learning-based spine segmentation.^20^

### Deep learning algorithm development for epicardial adipose tissue segmentation

We developed a deep learning system for fully automated EAT segmentation in NCCT images. The system consists of two stages (*Figure 1B*): (i) segmentation of the pericardium and (ii) identification of EAT within the pericardium. For pericardium segmentation, we implemented a UNet-family architecture known as UNet++^21^ using the PyTorch framework (version 1.8.1). UNet++ extends the classic UNet architecture by introducing dense skip pathways between encoder and decoder layers, enabling improved multi-scale feature learning and better capture of both global and local image context (*Figure 1C*).

We employed a 3D adaptation of UNet++ to perform voxel-level segmentation on volumetric NCCT images. The network comprises a five-level encoder–decoder architecture with nested dense skip connections for enhanced multi-scale feature fusion. Each convolutional block includes 3D convolutions, instance normalization, and Leaky ReLU activation (negative slope = 0.1). The encoder feature channel sizes were set to [32, 32, 64, 128, 256], with a final upsampling feature size of 32. Upsampling was performed using transposed convolutions, and feature maps from multiple previous levels were concatenated to construct the nested skip connections. While following the key principles of the original UNet++ design, our implementation extends it to support 3D volumetric inputs for accurate voxel-level segmentation.

Training was conducted on NCCT scans resampled to a voxel spacing of 1.6 × 1.6 × 1.6 mm and resized to 128 × 128 × 128 voxel cubes through cropping or padding. The model was optimized using Root Mean Square Propagation (RMSProp) to minimize the binary cross-entropy loss. Random weight initialization was used, with a fixed learning rate of 1e-4 and no learning rate scheduler. A mini-batch size of 1 was used. Training and validation loss curves were monitored after each run to evaluate convergence and detect potential overfitting.

Finally, to obtain EAT segmentation, the system automatically identified voxels with HU values between −190 and −30 from the original high-resolution image, constrained within the heart mask defined by the pericardium segmentation model. The EAT volume was computed by summing all identified EAT voxels and multiplying the total by the volume of a single voxel.

Model development and evaluation were performed on a Linux workstation equipped with an Intel Xeon 12-core processor, 256 GB RAM, 1 TB storage, and an NVIDIA GeForce RTX 3090 GPU with 24 GB of memory. Using this setup, the system required <30 s on average to perform EAT segmentation for a single scan.

### Statistical analysis

Continuous variables were reported as mean ± standard deviation (SD) or median (interquartile range), while categorical variables were presented as absolute and relative frequencies. Model performance was evaluated using the EAT relative volume error (%), defined as follows:


$$\mathrm{Relative}\;V_{EAT}\;\mathrm{Error}\;(\text{\%})=\frac{100\times |V_{EAT,\;predicted}-V_{EAT,\;reference}|}{V_{EAT,\;reference}}$$

Additionally, the Dice similarity coefficient (DSC) was calculated to assess the agreement between the automated EAT segmentation and the ground truth (manual segmentation). The DSC was computed using the following equation:


$$\mathrm{DSC}=\frac{2\times \mathrm{Intersection}}{\left(\mathrm{Prediction})\cup (\mathrm{Ground}\;\mathrm{Truth}\right)}$$

where ‘Intersection’ represents the overlapping area between prediction and ground truth.

The agreement between automatically derived EAT volume and those manually measured was also assessed using the Pearson correlation and Bland–Altman analysis. Logistic regression and receiver operating characteristic (ROC) curve analysis were used to evaluate the clinical utility of AI-derived EAT volume in predicting obstructive CAD. When examining associations with risk factors such as diabetes, hypertension, or hyperlipidaemia, EAT volume was normalized to body surface area (BSA) to account for individual differences in body size and anatomy. For analyses of obstructive CAD, absolute EAT volume was used, and multivariable models included adjustment for BMI to control for overall adiposity. Statistical significance was set at *P*  *<* 0.05.

## Results

### Population characteristics

The demographics and clinical risk factors for patient cohorts used in the training, internal validation, and external testing are shown in *Table 1*. Respective mean ages (±SD) of the three patient cohorts were 57 ± 10, 59 ± 11, and 60 ± 9 years. Individuals of Chinese ethnicity comprised the majority in the training and internal validation cohorts, while Caucasians and other non-Asian individuals accounted for 56% of the external validation cohort. In the combined internal and external testing populations, EAT volume was significantly associated with BMI (*r* = 0.41; *P* < 0.0001).

**Table 1:** Baseline characteristics of study

	Training cohort (*n* = 993)	Internal validation cohort (*n* = 250)	External validation cohort (*n* = 160)^a^
Age, years	57 ± 10	59 ± 11	60 ± 9
Male, *n* (%)	649 (65)	164 (66)	121 (86)
Body mass index, kg/m^2^	26 ± 5	26 ± 4	28 ± 4
Race^b^			
Chinese, *n* (%)	703 (71)	180 (72)	42 (26)
Malay, *n* (%)	64 (6)	16 (6)	11 (7)
Indian, *n* (%)	106 (11)	28 (11)	14 (9)
Caucasian, *n* (%)	8 (1)	5 (2)	70 (44)
Other non-Asian, *n* (%)	0 (0)	0 (0)	20 (13)
Others, *n* (%)	112 (11)	21 (9)	3 (2)
Hypertension, *n* (%)	518 (52)	121 (48)	67 (48)
Diabetes mellitus, *n* (%)	193 (19)	40 (16)	25 (18)
Hyperlipidaemia, *n* (%)	663 (67)	143 (57)	64 (46)
Smoking, *n* (%)	151 (15)	30 (12)	30 (21)
Family history, *n* (%)	408 (41)	89 (36)	37 (26)
CAC score^c^	51 (2, 230)	89 (16, 309)	—

The training and internal validation cohorts were derived from three local centres, while the external validation cohort consisted of data from multiple countries and centres.

Data are presented as mean ± SD, median (interquartile range), or number (%).

^a^Data on age, sex, body mass index, and prevalence of hypertension, diabetes, hyperlipidaemia, smoking, and family history of cardiovascular disease were available for 140 patients. These parameters were not available for the 20 patients from the Cardiac Fat Database—Computed Tomography.

^b^Race was summarized for all 160 patients, as it was the only available parameter for the 20 patients from the Cardiac Fat Database—Computed Tomography. These 20 patients were all of non-Asian race and were categorized under ‘Other non-Asian.’

^c^Coronary artery calcium (CAC) score was not available in the external validation cohort.

### Inter-reader repeatability for epicardial adipose tissue volume

Inter-observer variability was assessed in 50 randomly selected cases. The average DSC for pericardium segmentation between the two analysts was 0.968 ± 0.028. The Pearson correlation coefficient for EAT volume between analysts was excellent (*r* = 0.992; *P* < 0.0001), with a non-significant mean bias of −1.0 cm^3^ (*P* = 0.522; Bland–Altman 95% limits of agreement: −13.8, 11.8 cm^3^; see Supplementary material online, *Figure S4*). The intra-class correlation coefficient for EAT volume was 0.996 [95% confidence interval (CI): 0.993, 0.998].

### Internal validation of the model

We recorded the time required for manual annotation and for the deep learning-based EAT segmentation in a subset of the testing cohort (*n* = 150). The mean ± SD time for manual segmentation was 20.2 ± 3.6 min per scan, while that for the deep learning method was 27.0 ± 11.7 s per scan. The distribution of segmentation times for both manual and automated methods is shown as frequency (histogram) plots in Supplementary material online, *Figures S5A*–*B*. A direct comparison of the time required for manual vs. deep learning segmentation is presented in Supplementary material online, *Figure S5C*.

**Figure 2:**
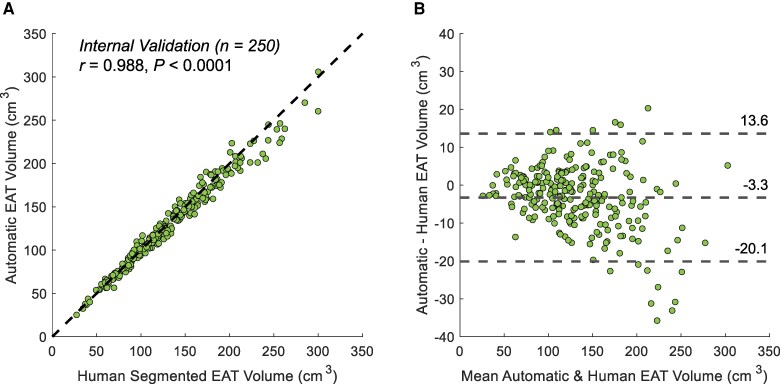
Internal validation in 250 cases. (*A*) Correlation plot and (*B*) Bland–Altman plot comparing automated and expert manual epicardial adipose tissue volume measurements. EAT, epicardial adipose tissue.

**Figure 3:**
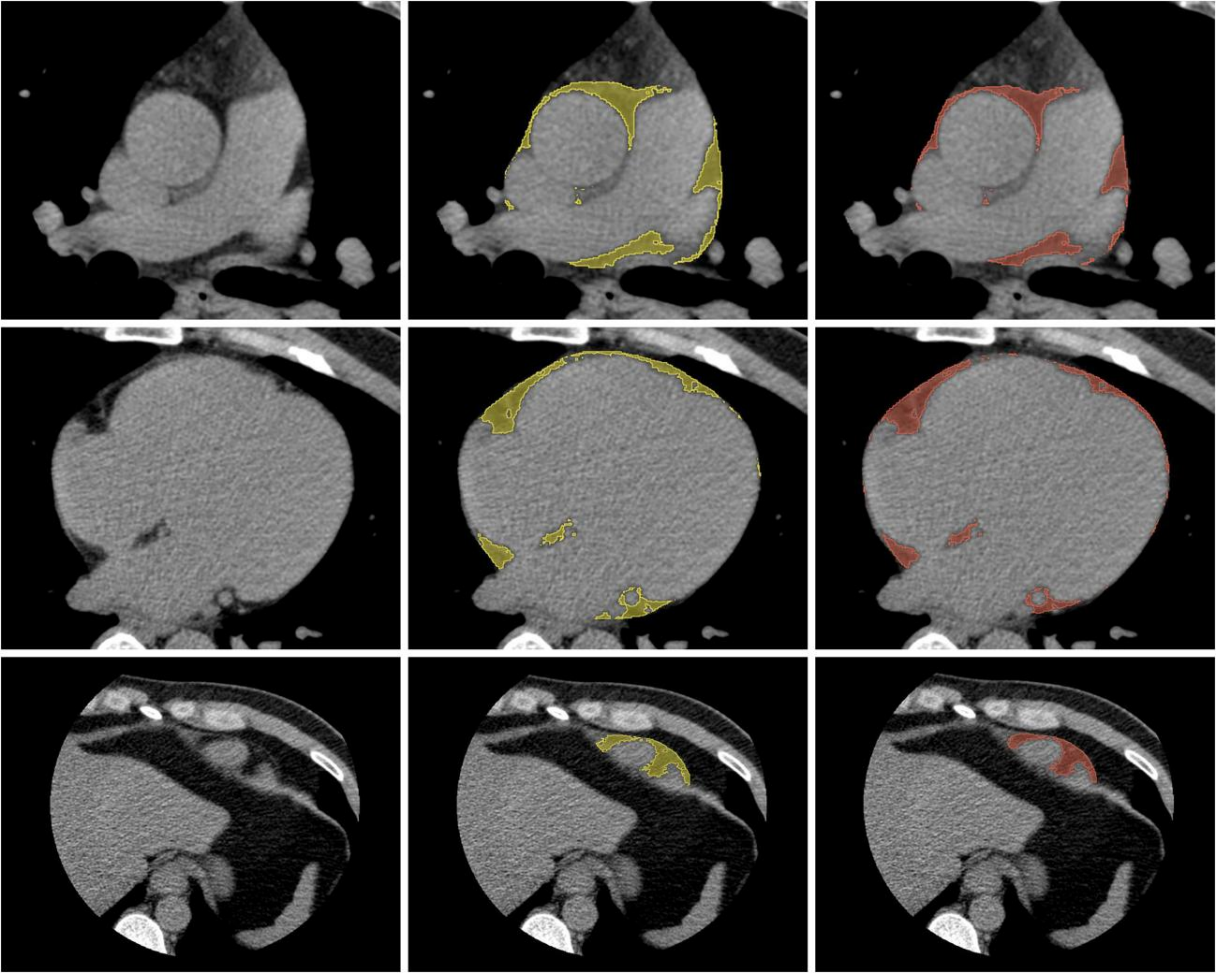
Comparison of human annotations (middle column) and AI predictions (right column) for epicardial adipose tissue at the superior limit of the heart (top row), the mid-ventricular slice (middle row), and the inferior limit of the heart (bottom row). The left column shows the corresponding non-contrast computed tomography images.

*Figure 2A* shows the correlation between EAT volume measurements by the AI model and an expert reader in the internal validation dataset of 250 patient scans (Pearson correlation coefficient *r* = 0.988; *P* < 0.0001). The Bland–Altman analysis (*Figure 2B*) demonstrated that the average difference in EAT volume quantification between AI and the reader was −3.3 cm^3^ (bias), with limits of agreement −20.1 and 13.6 cm^3^. The average relative EAT volume error was 4.84 ± 3.53%, and the average 3D DSC was 0.922 ± 0.019. Representative examples of manual annotations and AI-predicted EAT segmentations at the superior, middle, and inferior levels of the heart are shown in *Figure 3*. Performance of the deep learning model across different centres is summarized in *Table 2*. Centre-specific Bland–Altman plots are provided in the Supplementary material (Supplementary material online, *Figure S6*).

**Table 2:** Comparison of the deep learning model performance for each of the centres

Centre (number of internal validation patients)	Performance metrics					
	Relative EAT volume error, %		Mean EAT DSC		Bland–Altman statistics, cm^3^	
	Mean	Worst	Mean	Worst	Bias	Limits of agreement
All (250)	4.84 ± 3.53	19.67	0.922 ± 0.019	0.844	−3.28	[−20.11, 13.56]
Centre 1 (150)	5.17 ± 3.72	19.67	0.922 ± 0.017	0.867	−4.61	[−22.17, 12.96]
Centre 2 (50)	3.48 ± 2.87	10.84	0.924 ± 0.023	0.844	0.85	[−8.95, 10.65]
Centre 3 (50)	4.83 ± 3.20	13.49	0.918 ± 0.022	0.858	−2.47	[−20.77, 15.82]

Data are presented as mean ± SD.

EAT, epicardial adipose tissue; DSC, Dice similarity coefficient.

To assess the potential influence of scanner vendors on segmentation performance, we conducted additional analyses comparing model accuracy across the CT scanner vendors in our dataset. The results showed no significant differences in performance metrics across vendors (one-way ANOVA: *P* = 0.173 for EAT relative volume prediction error). We also examined other scanner-related factors, including tube current (mA), tube current–time product (mAs), and radiation dose. Spearman correlations between EAT relative volume prediction error and these factors were ρ = −0.041 for tube current, −0.057 for tube current–time product, and 0.063 for radiation dose, all with *P* > 0.10. Similar results were observed for the DSC, with no significant differences across vendors and no significant correlation with scanner-related parameters.

### External validation of the model

**Figure 4:**
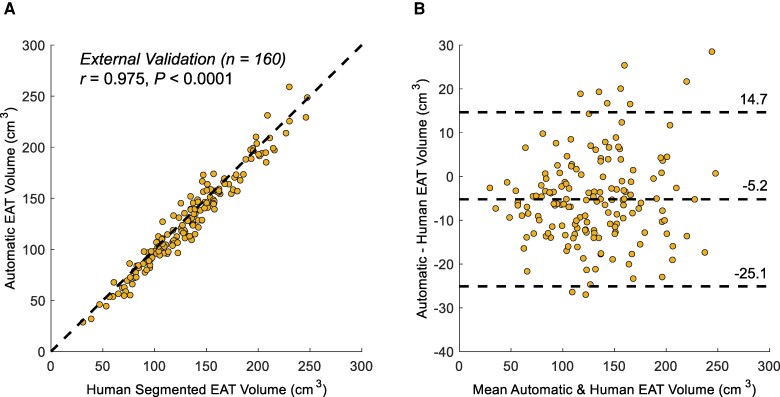
External validation in 160 cases. (*A*) Correlation plot and (*B*) Bland–Altman plot comparing automated and expert manual epicardial adipose tissue volume measurements. EAT, epicardial adipose tissue.

The final deep learning model was evaluated on the external validation cohort (*n* = 160). The mean EAT volume was 133 ± 45 cm^3^ by manual annotation and 128 ± 46 cm^3^ by the AI model (*P* = 0.305). The Pearson correlation between the AI-predicted and expert-annotated EAT volumes was excellent (*r* = 0.975; *P* < 0.0001; *Figure 4A*). The Bland–Altman analysis (*Figure 4B*) showed a mean bias of −5.2 cm^3^, with 95% limits of agreement ranging from −25.1 to 14.7 cm^3^. The mean DSC was 0.876 ± 0.021.

Among these 160 cases, 90 were from non-Asian individuals. In this subgroup, the Pearson correlation remained excellent (*r* = 0.970; *P* < 0.0001; Supplementary material online, *Figure S7A*), with a mean bias of −3.2 cm^3^ and 95% limits of agreement from −25.1 to 18.7 cm^3^ (see Supplementary material online, *Figure S7B*). Model performance was comparable between non-Asian and Asian subgroups.

### Association of epicardial adipose tissue with cardiovascular risk factors

In the combined internal and external testing cohorts, the mean BSA-indexed EAT volume quantified by the automated algorithm was 71 ± 25 cm^3^/m^2^. Epicardial adipose tissue volume index was positively correlated with age (*r* = 0.320; *P* < 0.0001); each additional year of age was associated with a 0.60 cm^3^/m^2^ increase in EAT volume index after adjusting for confounders. In multivariable analysis adjusted for age, sex, and other cardiovascular risk factors, both hypertension and diabetes mellitus were independently associated with higher EAT volume index. Patients with hypertension had a mean EAT volume index 5.8 cm^3^/m^2^ higher than those without hypertension (95% CI: 0.4, 11.1; *P* = 0.035), and those with diabetes had a mean increase of 8.3 cm^3^/m^2^ compared with non-diabetic patients (95% CI: 1.5, 15.0; *P* = 0.017).

### Association of epicardial adipose tissue with obstructive coronary artery disease

In the internal test dataset (where obstructive CAD assessment was available), univariate logistic regression analyses identified several variables significantly associated with the presence of obstructive CAD, defined as ≥50% stenosis. Increasing EAT volume was associated with higher odds of obstructive CAD [odds ratio (OR) per 10 cm^3^, 1.11; 95% CI, 1.05, 1.18; *P* < 0.0001], as were older age (OR per 10 years, 1.75; 95% CI, 1.36–2.26; *P* < 0.0001), presence of diabetes (OR, 2.48; 95% CI, 1.22, 5.03; *P* = 0.012), coronary artery calcium (CAC) score per 100 Agatston units (OR, 1.80; 95% CI, 1.49, 2.17; *P* < 0.0001), and presence of hypertension (OR, 2.40; 95% CI, 1.42, 4.05; *P* = 0.001). In multivariable logistic regression, EAT volume (OR per 10 cm^3^, 1.11; 95% CI, 1.04, 1.19; *P* = 0.004) and CAC score (OR per 100 units, 1.92; 95% CI, 1.54, 2.38; *P* < 0.0001) remained independent predictors of obstructive CAD (*Table 3*). The global χ^2^ statistic increased from 81.7 with coronary calcium score alone to 93.3 when EAT volume was added (*P* = 0.001), indicating improved risk prediction. Receiver operating characteristic curve analysis identified an optimal EAT volume threshold of 140 cm^3^ for predicting obstructive CAD (area under the curve, 0.632; sensitivity, 49.2%; specificity, 72.3%; Supplementary material online, *Figure S8*).

**Table 3:** Univariate and multivariable logistic regression analysis of predictors related to the presence of obstructive CAD (≥50% stenosis)

Variable	Univariate		Multivariable	
	OR (95% CI)	*P* value	OR (95% CI)	*P* value
Age (per 10 years)	1.75 (1.36–2.26)	<0.0001	—	—
Sex (male)	1.46 (0.86–2.47)	0.160	—	—
Body mass index, kg/m^2^	1.04 (0.97–1.10)	0.269	—	—
Hypertension	2.40 (1.42–4.05)	0.001	—	—
Diabetes mellitus	2.48 (1.22–5.03)	0.012	—	—
Hyperlipidaemia	1.58 (0.93–2.68)	0.091	—	—
Smoking	0.53 (0.24–1.19)	0.123	—	—
Family history	1.04 (0.61–1.76)	0.889	—	—
EAT volume (per 10 cm^3^)	1.11 (1.05–1.18)	<0.0001	1.11 (1.04–1.19)	0.004
CAC score (per 100 Agatston units)	1.80 (1.49–2.17)	<0.0001	1.92 (1.54–2.38)	<0.0001

CAD, coronary artery disease; EAT, epicardial adipose tissue; CAC, coronary artery calcium; OR, odds ratio; CI, confidence interval.

To ensure robustness, a sensitivity analysis was conducted using EAT volume indexed to BSA, with additional adjustment for BMI. The results remained consistent: EAT volume index was significantly associated with obstructive CAD (OR, 1.21; 95% CI, 1.06, 1.38; *P* = 0.006; Supplementary material online, *Table S2*).

## Discussion

In this study, we developed and validated a deep learning model for fast, automated, and accurate quantification of EAT from routine coronary calcium scoring CT scans, using a large, multi-centre, pan-Asian dataset. The model demonstrated excellent correlation and agreement with expert manual annotations while reducing EAT quantification time from 20–30 min to ∼30 s. This approach can be integrated into clinical software, potentially alleviating clinician workload and enabling routine assessment of EAT within standard clinical workflows. The model was externally validated on an independent cohort that included non-Asian patients and achieved similar performance, demonstrating strong agreement with expert observers and supporting its generalizability across diverse populations. Furthermore, AI-derived EAT volume was significantly associated with established cardiovascular risk factors, including hypertension and diabetes, and remained independently associated with obstructive CAD after adjustment for age, sex, and other confounders.

The volume of cardiac EAT has increasingly been recognized as a valuable biomarker for predicting the risk of CAD.^3^ Routine non-contrast coronary calcium scoring CT scans are the preferred imaging modality for visualizing and quantifying EAT.^22^ However, one of the primary barriers to its widespread clinical adoption is the lack of a reliable, timely, and fully automated quantification method.^7^ Early approaches required labour-intensive manual contouring of the pericardium—a process that was both time-consuming and subject to inter-observer variability. This was later improved by semi-automated segmentation techniques, which allowed expert readers to make manual adjustments to enhance accuracy.^8,9,23,24^ More recently, the application of AI, particularly deep learning, has paved the way for rapid and fully automated EAT segmentation,^10,25–28^ potentially streamlining clinical applications.

In this study, our AI model was implemented using UNet++ architecture, with two key innovations. First, we designed a dedicated preprocessing strategy with an additional HU-based threshold class in the inferior region of the scan. The rationale was to introduce a separate class label in regions where the AI model consistently struggled—particularly in the inferior (apical) slices—due to the subtle contrast and lack of clear anatomical boundaries. This redundant class helped the model learn to better differentiate between background, EAT, and the pericardial boundaries. This strategy is conceptually similar to the approach described by Vania *et al*.,^20^ where redundant class labels were introduced around the spine structure to improve boundary delineation and reduce misclassification with surrounding anatomy (e.g. ribs). By assigning different class values to regions adjacent to the main segmentation target, the model is penalized for boundary errors and learns more robust features. Likewise, in our work, the redundant fat-density class in the inferior pericardial region acts as an intermediate zone that guides the model to better identify and preserve the true pericardial boundary. Comparative analysis confirmed the effectiveness of this strategy, showing consistent improvements in segmentation performance, including increased DSC and reduced relative EAT volume error. Second, our method incorporates 3D voxel-level processing to leverage volumetric context, which distinguishes it from many prior studies that rely primarily on 2D convolutional neural networks applied to individual axial slices.^29–32^ By incorporating 3D contextual information, our method captures spatial continuity and anatomical coherence more effectively. This 3D strategy also led to measurable performance gains, with improvements in both DSC and volume estimation accuracy.

We compared our model to existing works in the literature (see Supplementary material online, *Table S3*).^10,11,25,28–36^ Our model achieved the highest reported Pearson correlation coefficient for EAT volume estimation. Additionally, the number of patient scans used for training and validation in our study is among the highest, compared with other studies. Notably, when disclosed, most existing studies on CAD prediction models and imaging biomarkers have predominantly involved populations of White or European ethnicity.^10,25^ In contrast, our model was developed using a pan-Asian population representative of Singapore’s diverse ethnic makeup, including Chinese, Malay, Indian, and other Asian subgroups. This provides a unique opportunity to explore CAD risk and its underlying mechanisms in a more ethnically diverse and often underrepresented population. Ethnic differences in body composition and cardiovascular risk factors are increasingly recognized as clinically relevant. For example, Asians are known to have higher levels of visceral adiposity—including EAT—compared with Caucasians at similar BMI levels.^12,37^ This disproportionately higher visceral fat burden contributes to a greater cardiometabolic risk in Asians, even among individuals classified as non-obese by conventional BMI thresholds.^13,38^ As such, findings derived from European-centric populations may not be directly generalizable to Asian populations. Our pan-Asian model aims to address this gap by providing insights that are more directly applicable to clinical risk stratification and management in Asian individuals, particularly given the higher prevalence of metabolically active fat depots like EAT, which have been independently associated with obstructive CAD.

The observed limits of agreement in the internal test dataset were −20.1–13.6 cm^3^, corresponding to ∼−16 to 11% relative to the mean EAT volume of 127 cm^3^. While this range reflects some degree of case-level variability in the actual measured EAT volumes, the relative EAT volume error was low at 4.84 ± 3.53%, indicating strong agreement with minimal bias. The limits of agreement are consistent with or narrower than those reported in prior studies. For example, Miller *et al*.^25^ reported limits ranging from −46.1–36.7 cm^3^, Qu *et al*.^31^ reported −25–21 cm^3^, Abdulkareem *et al*.^29^ reported −22.7–17.1 cm^3^, Hoori *et al*.^11^ reported −17.0–20.0 cm^3^, and Commandeur *et al*.^10^ reported −19.6–21.4 cm^3^ (see Supplementary material online, *Table S3*). These comparisons indicate that our method achieves an agreement comparable to, or better than, existing approaches. Additionally, the magnitude of these limits is in line with the inter-rater variability typically reported for manual or semi-automated EAT segmentation—both in our study and the broader literature (−8.54–25.13 cm^3^ in Commandeur *et al*.,^10^ −14.1–17.9 cm^3^ in Hoori *et al*.^11^). This suggests that our AI method performs on par with expert readers in terms of reproducibility, supporting its acceptability for clinical and research applications. Importantly, despite this variation, our method demonstrated good discriminatory performance in ROC analysis and remained a significant predictor of obstructive CAD in both univariate and multivariable models. This suggests that while absolute agreement has some inherent variability, the clinical utility of the automated measurement is preserved.

Model performance was validated through both internal and external validation cohorts, demonstrating strong robustness across diverse datasets. Within the internal validation cohort, subgroup analyses across participating heart centres revealed no significant differences in relative EAT volume error or DSC, indicating consistent and reliable model performance across institutions. To further assess generalizability across populations, we evaluated the model on an external validation cohort that included a substantial proportion of non-Asian individuals—90 out of 160 patients. Model performance in the non-Asian subgroup remained comparable to that in the Asian subgroup, supporting the model’s robustness across different ethnic and geographic populations.

The findings of this study highlight a significant association between EAT and key cardiovascular risk factors, including age, hypertension, and diabetes mellitus. The observed positive correlation between EAT volume index and age suggests a progressive accumulation of EAT with advancing age, consistent with prior studies linking visceral adiposity to age-related metabolic and cardiovascular changes.^39^ After adjusting for potential confounders, both hypertension and diabetes remained independently associated with increased EAT volume index, reinforcing the role of EAT as a marker of cardiometabolic risk.^10,26^ These results support the hypothesis that EAT may contribute to the pathophysiology of cardiovascular disease through local pro-inflammatory and metabolic effects,^40^ emphasizing its potential utility in risk stratification.

In addition, our findings demonstrate a significant association between EAT volume and obstructive CAD, further supporting the role of EAT as a biomarker for coronary atherosclerosis.^41^ Patients with obstructive CAD exhibited significantly higher EAT volumes compared with those without, suggesting a potential link between increased EAT deposition and the progression of coronary atherosclerosis. Logistic regression analysis confirmed this association, with EAT volume remaining an independent predictor of obstructive CAD after adjusting for age, sex, and other cardiovascular risk factors. Given that EAT is metabolically active and anatomically adjacent to the coronary vasculature,^42^ its expansion may reflect underlying pathological processes contributing to CAD. Integrating EAT quantification with other clinical and imaging-based risk factors could enhance predictive models, improving early detection and risk stratification for obstructive CAD.

This study has several limitations. First, as part of an ongoing prospective trial, long-term clinical outcomes for CAD patients with quantified EAT volume are not yet available. Future analyses will be essential to determine whether EAT volume independently predicts adverse cardiovascular events, as the ultimate goal is to establish EAT as a biomarker for enhanced CAD risk stratification. Second, although our findings underscore a strong association between EAT and cardiovascular risk factors, further research is needed to elucidate the underlying biological mechanisms and assess whether interventions aimed at reducing EAT can lead to improved cardiovascular outcomes. Third, specific body or chest phenotypes (e.g. body habitus and thoracic dimensions) were not available in our dataset. Incorporating such phenotypic information in future studies could provide valuable insights into anatomical variability that may affect segmentation accuracy. Fourth, waist circumference was not collected as part of the imaging study protocol. Future studies with complete anthropometric data, including waist circumference, will be necessary to validate our findings and further clarify the independent contribution of EAT volume. Finally, longitudinal studies are warranted to evaluate whether temporal changes in EAT volume correlate with disease progression and clinical events, which would further clarify its utility as a prognostic marker.

In conclusion, we developed an automated system for EAT volume quantification using a deep learning-based approach and comprehensively validated its performance against expert human assessments. The results demonstrate strong accuracy and reliability, supporting its potential for clinical implementation. Ongoing efforts are underway to integrate the system as a software solution within clinical workflows. This advancement holds substantial promise for improving the efficiency and consistency of EAT assessment, ultimately contributing to enhanced cardiovascular risk evaluation and patient care.

## Lead author biography


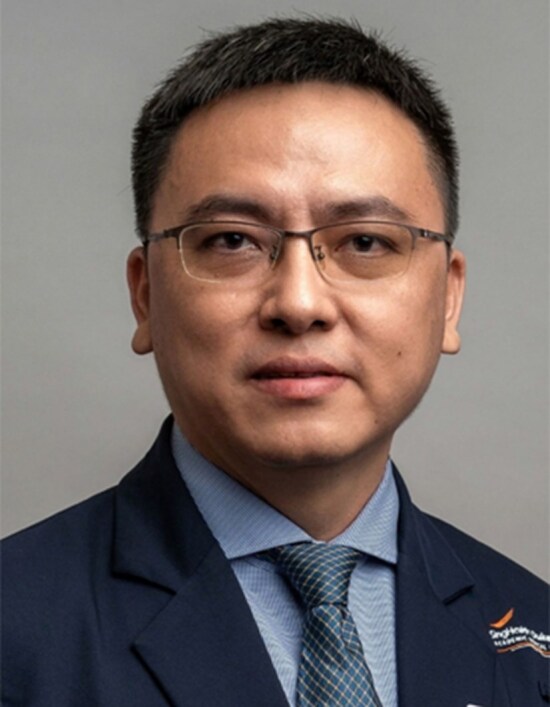


Dr Shuang Leng is affiliated with the National Heart Centre Singapore and Duke-NUS Medical School Singapore. His expertise lies in cardiac image processing and computer science, with research interests spanning the analysis of cardiac MR, CT, and 3D echocardiographic images. He focuses on algorithm development for cardiac segmentation and the quantification of regional contractile function and myocardial strain, as well as the application of artificial intelligence in cardiovascular image analysis.

## Supplementary Material

ztaf116_Supplementary_Data

## Data Availability

The data underlying this article are available in the article and in its online Supplementary material.
