# Supplementary material for: Deep learning-based quantification of epicardial adipose tissue volume from non-contrast computed tomography images: a multi-centre study
Source: Eur Heart J Digit Health. 2025 Oct 13;6(6):1223–33. doi: 10.1093/ehjdh/ztaf116 (PMC12629654; doi:10.1093/ehjdh/ztaf116)
Supplement: ztaf116_Supplementary_Data [file ztaf116_supplementary_data.docx]

**Supplementary Material**

**Supplementary Table 1.** Typical scanning parameters.

| **CT scanner** | **Tube voltage, kV** | **Slice thickness, mm** | **Pixel size** | **Typical pixel spacing, mm** |
| --- | --- | --- | --- | --- |
| Toshiba Aquilion ONE | 120 | 3 | 512×512 | 0.376×0.376 |
| Siemens 384-slice dual source Somatom Force | 120 | 3 | 512×512 | 0.354×0.354 |
| Siemens 256-slice dual source Somatom Definition Flash | 120 | 3 | 512×512 | 0.307×0.307 |

**Supplementary Table 2.** Univariate and multivariable logistic regression analyses of predictors, including EAT volume index normalized to body surface area, associated with the presence of obstructive CAD defined as ≥50% stenosis.

| **Variable** | **Univariate** | | **Multivariable** | |
| --- | --- | --- | --- | --- |
|  | OR (95% CI) | *P* value | OR (95% CI) | *P* value |
| **Age (per 10 years)** | 1.75 (1.36-2.26) | <0.0001 | - | - |
| **Sex (male)** | 1.46 (0.86-2.47) | 0.160 | - | - |
| **Body mass index, kg/m^2^** | 1.04 (0.97-1.10) | 0.269 | - | - |
| **Hypertension** | 2.40 (1.42-4.05) | 0.001 | - | - |
| **Diabetes mellitus** | 2.48 (1.22-5.03) | 0.012 | - | - |
| **Hyperlipidemia** | 1.58 (0.93-2.68) | 0.091 | - | - |
| **Smoking** | 0.53 (0.24-1.19) | 0.123 | - | - |
| **Family history** | 1.04 (0.61-1.76) | 0.889 | - | - |
| **EAT volume index**  **(per 10 cm^3^/m^2^)** | 1.18 (1.06-1.31) | 0.002 | 1.21 (1.06-1.38) | 0.006 |
| **CAC score**  **(per 100 Agatston units)** | 1.80 (1.49-2.17) | <0.0001 | 1.90 (1.53-2.35) | <0.0001 |

CAD, coronary artery disease; EAT, epicardial adipose tissue; CAC, coronary artery calcium; OR, odds ratio; CI, confidence interval.

**Supplementary Table 3.** Performance comparison with existing works in the literature.

| **Authors** | **Year** | **Number of patient scans** | **Ethnicity** | **Image** | **Model** | **EAT volume Pearson's *r*** | **Pericardium mean Dice score** | **EAT mean Dice score** | **Bland-Altman analysis - EAT volume**  **(bias [limits of agreement])** |
| --- | --- | --- | --- | --- | --- | --- | --- | --- | --- |
| Miller et al. [25] | 2024 | 8781 | Predominantly White | ungated CT | Convolutional LSTM model | 0.90 | - | - | -4.7 [-46.1, 36.7] |
| West et al. [28] | 2023 | 3720 | Multi-ethnic | CCTA | 3D UNet | 0.97 | - | - | 3.2 [-13.6, 17.2] |
| Abdulkareem et al. [29] | 2022 | 300 | Not disclosed (presumed White) | contrast CT | ResNet+, UNet | 0.98 | - | 0.844 | -2.8 [-22.7, 17.1] |
| Goncharov et al. [33] | 2022 | 569 | Not disclosed | chest CT | 3D UNet | 0.96 | 0.95 | - | - |
| Hoori et al. [11] | 2022 | 93 | Not disclosed (presumed White) | non-contrast CT | DeepLab-v3 plus | 0.98 | - | 0.89 | 1.5 [-17.0, 20.0] |
| Li et al. [30] | 2022 | 70 | Chinese, White | contrast and non-contrast CT | RDU-Net | 0.99 | - | 0.92 | - |
| Liu et al. [34] | 2023 | 154 | White | non-contrast CT | 3D UNet, 3D Attention UNet, DAU-Net, UNet++ | 0.94 | - | 0.80 | - |
| Qu et al. [31] | 2022 | 103 | Not disclosed (presumed Chinese) | non-contrast CT | CNN, UNet | 0.95 | - | 0.88 | 2.6 [-25, 21] |
| Molnar et al. [35] | 2021 | 1811 | Predominantly European | non-contrast CT | EAT-Net, Crop-Net | 0.95 | - | 0.90 | -1.8 [-13, 11] |
| Zhang et al. [32] | 2020 | 20 | White | non-contrast CT | dual U-Nets | 0.93 | - | 0.91 | - |
| He et al. [36] | 2020 | 200 | Not disclosed | non-contrast CT | 3D Deep attention UNet | - | - | 0.85 | -8.8 [-31.9, 14.3] |
| Commandeur et al. [10] | 2019 | 850 | Multi-ethnic, predominantly European | non-contrast CT | CNN | 0.97 | - | 0.873 | 0.5 [-19.6, 21.4] |
| **Current Study** | **2025** | **1403** | **Pan-Asian** | **non-contrast CT** | **3D UNet++, redundant class** | **0.98** | **0.96** | **0.922** | **-3.3 [-20.1, 13.6]** |


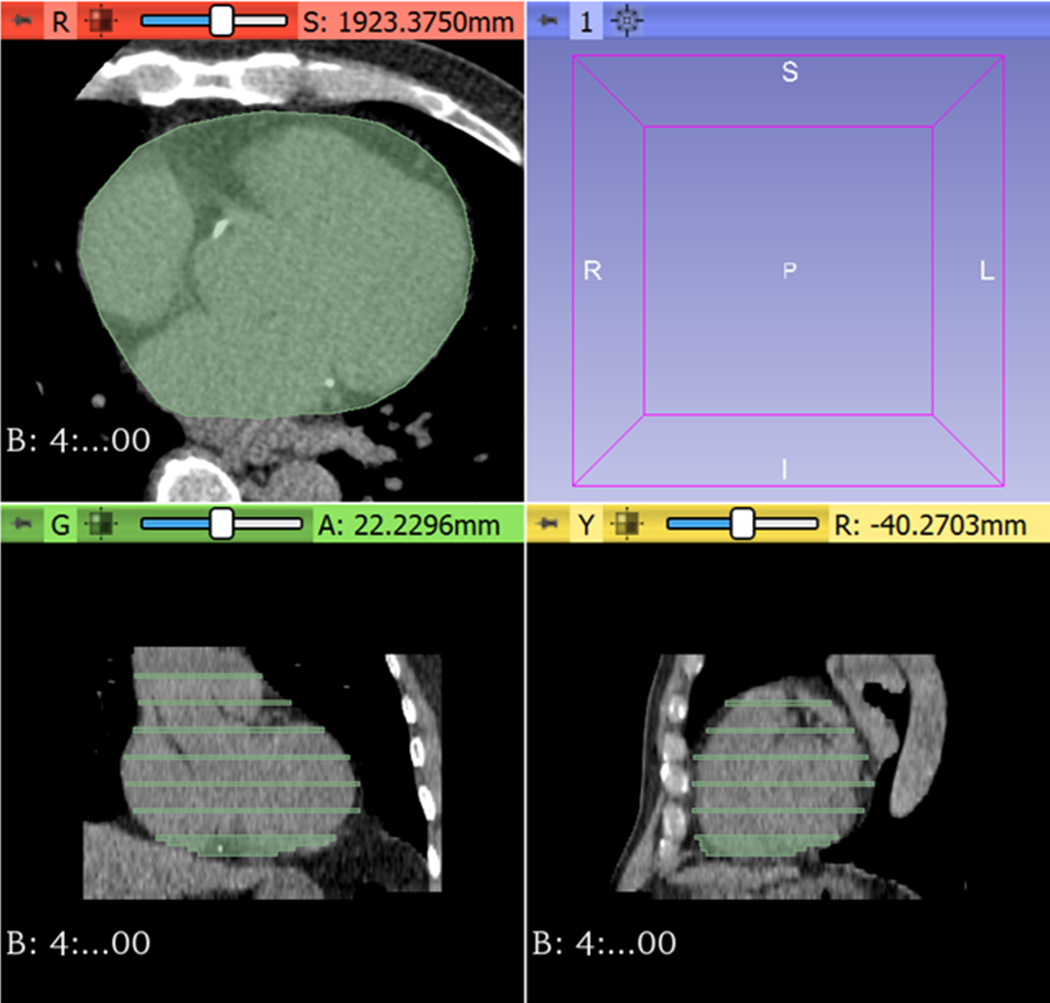


**Supplementary Figure 1.** Manual segmentation of epicardial adipose tissue using 3D Slicer. The panels display the axial (top-left), coronal (bottom-left), and sagittal (bottom-right) views of the heart. Annotators perform pericardium segmentation primarily on the axial view, using the coronal and sagittal views as references for anatomical accuracy.


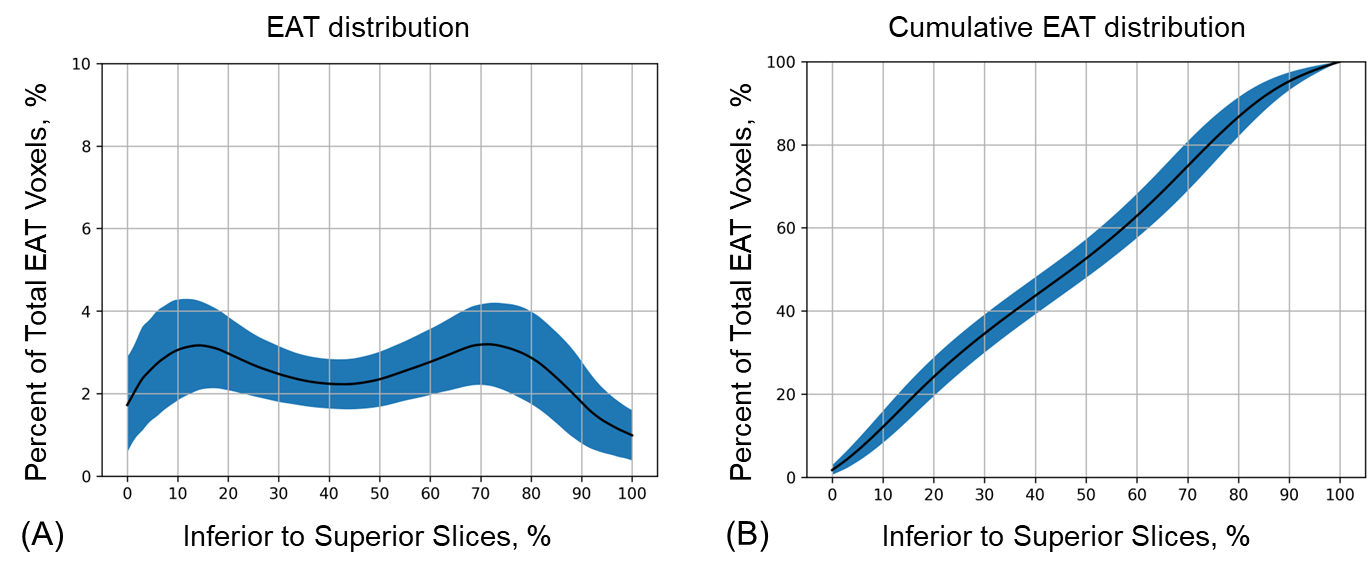


**Supplementary Figure 2.** (A) Distribution of epicardial adipose tissue (EAT) voxels across axial pericardial slices from inferior to superior, normalized to the total EAT voxel count across all slices. The black line indicates the mean percentage of EAT voxels per slice, with the shaded blue area representing ±1 standard deviation. (B) Cumulative EAT voxel count from inferior to superior, obtained by sequentially summing EAT voxels from the most inferior (apical) to the most superior (basal) pericardial slice, illustrating the progressive accumulation of total EAT volume.


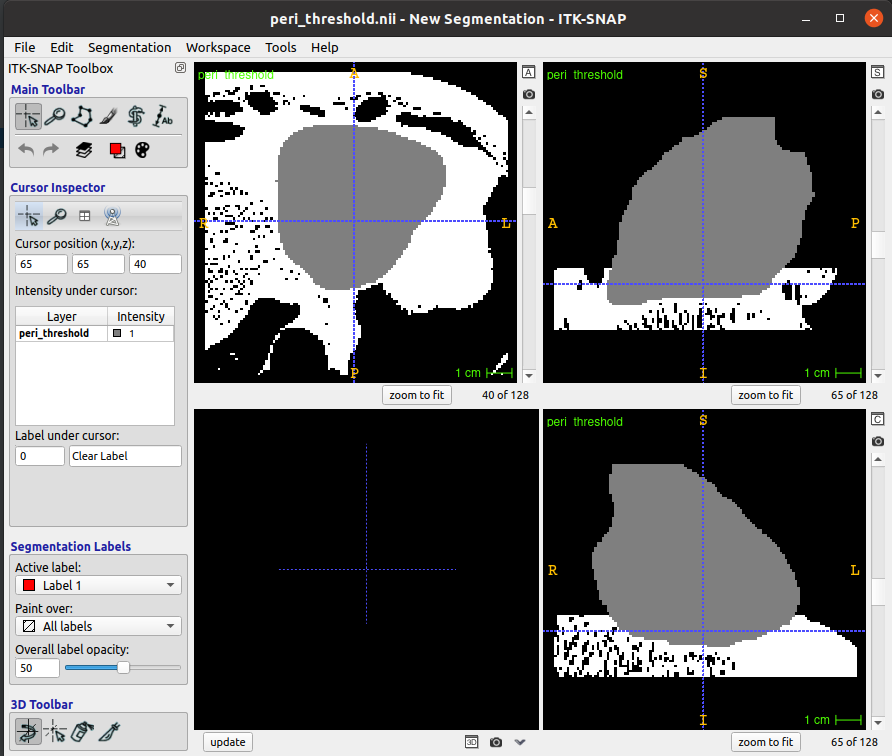


**Supplementary Figure 3.** Visualization of the added redundant class (white) alongside the pericardium (gray) and background (black) classes.


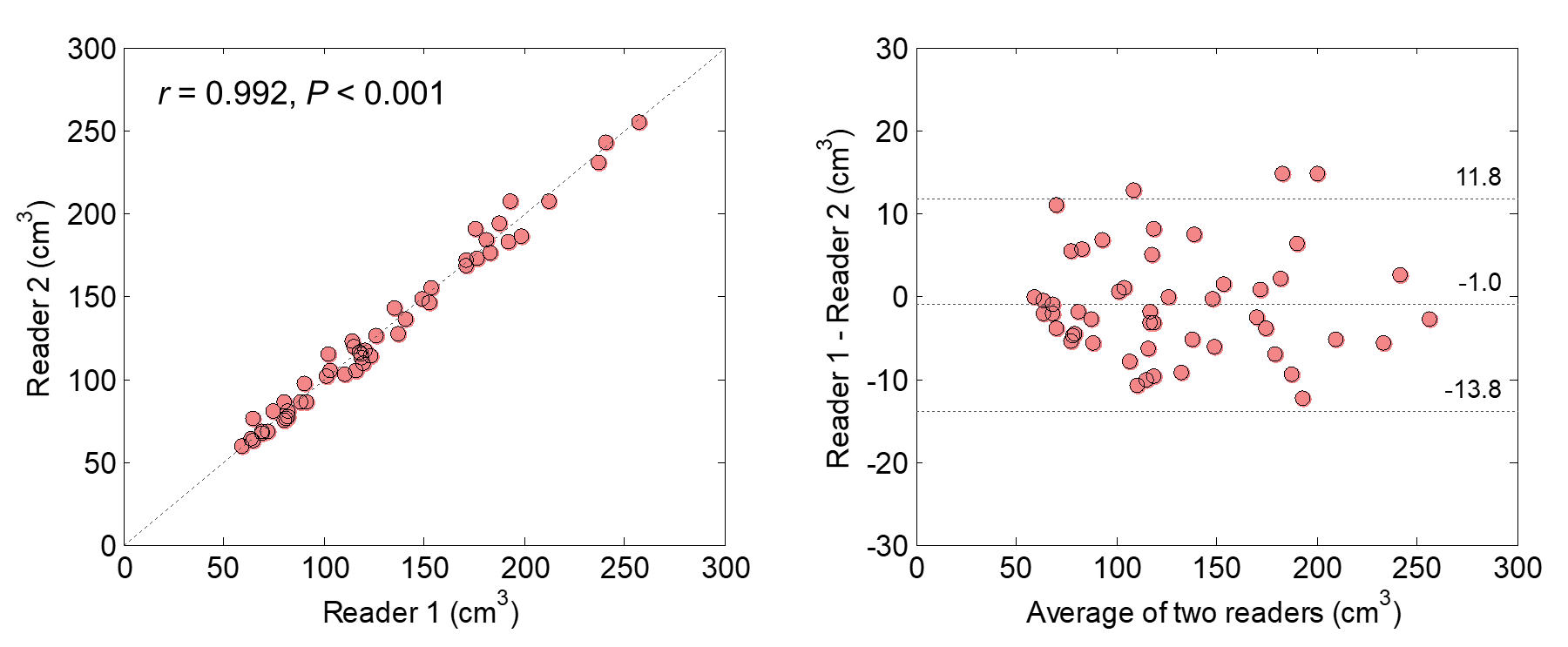


**Supplementary Figure 4.** Comparison of manually derived epicardial adipose tissue volumes by two independent readers in 50 cases. (Left) Correlation plot (Right) Bland-Altman plot.


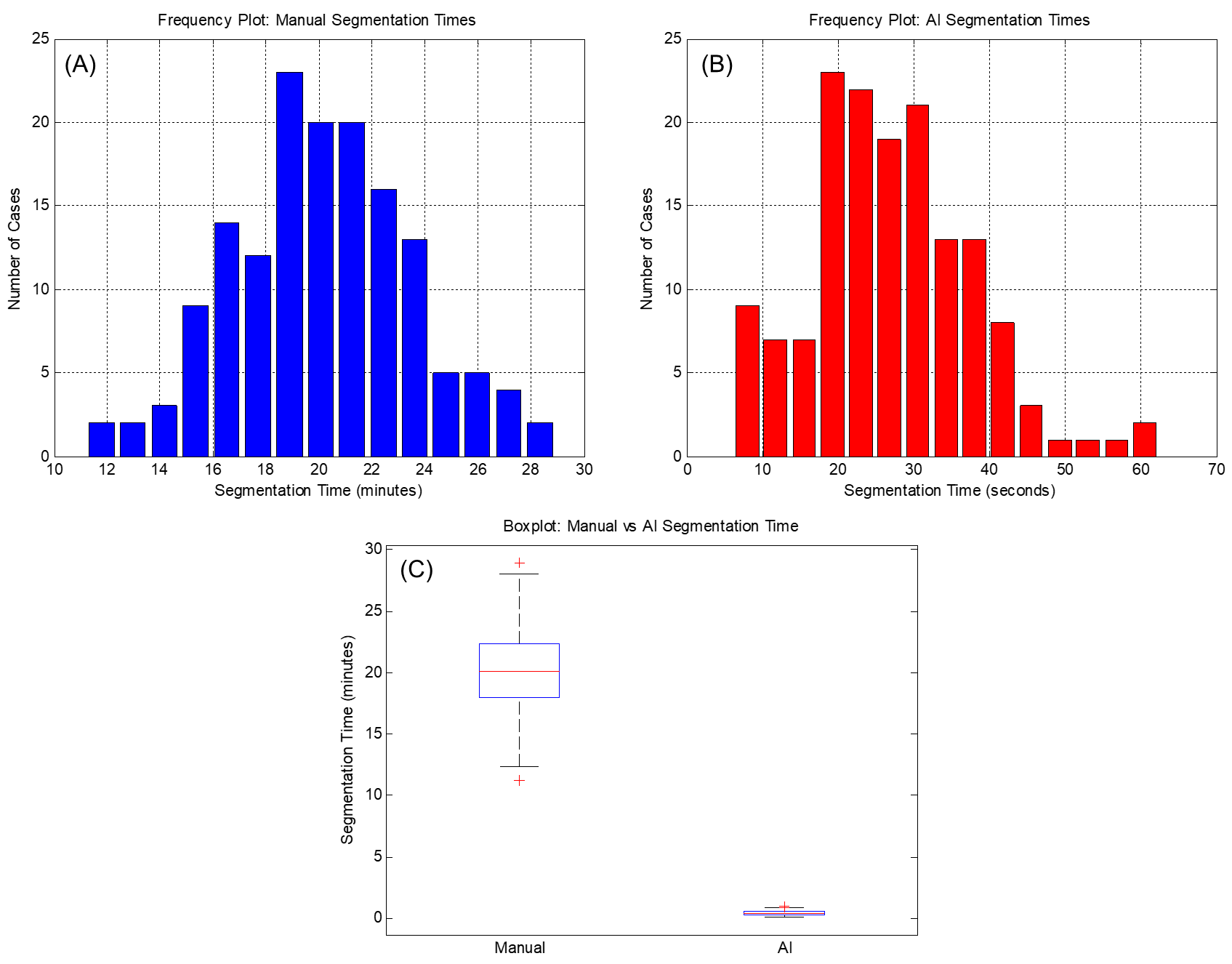


**Supplementary Figure 5.** (A) Distribution of segmentation times (**in minutes**) for manual method. (B) Distribution of segmentation times (**in seconds**) for automated method. (C) Direct comparison of the time required for manual versus deep learning segmentation.


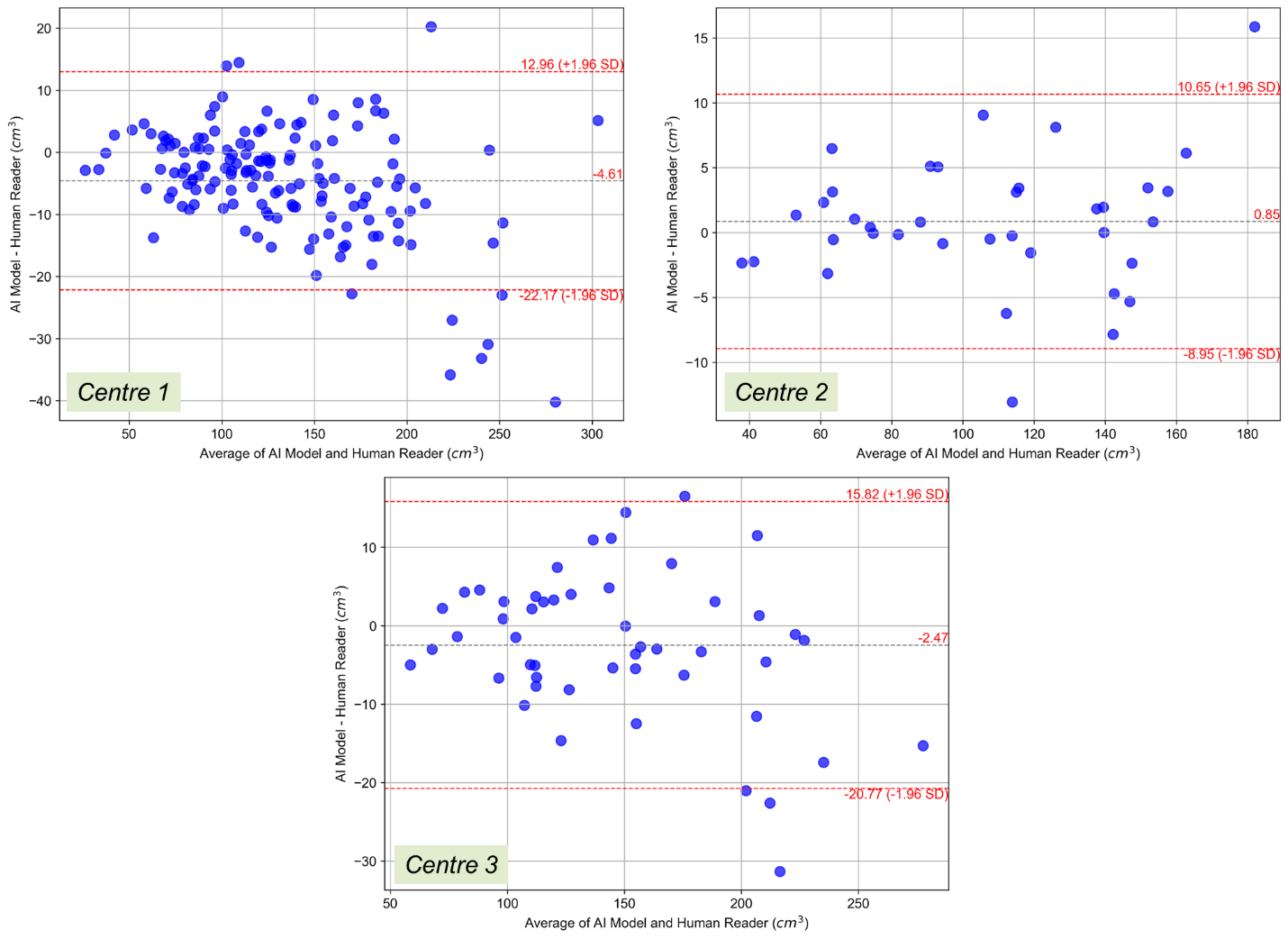


**Supplementary Figure 6.** Bland-Altman plots showing agreement between automated and expert manual EAT volume measurements for each participating centre.


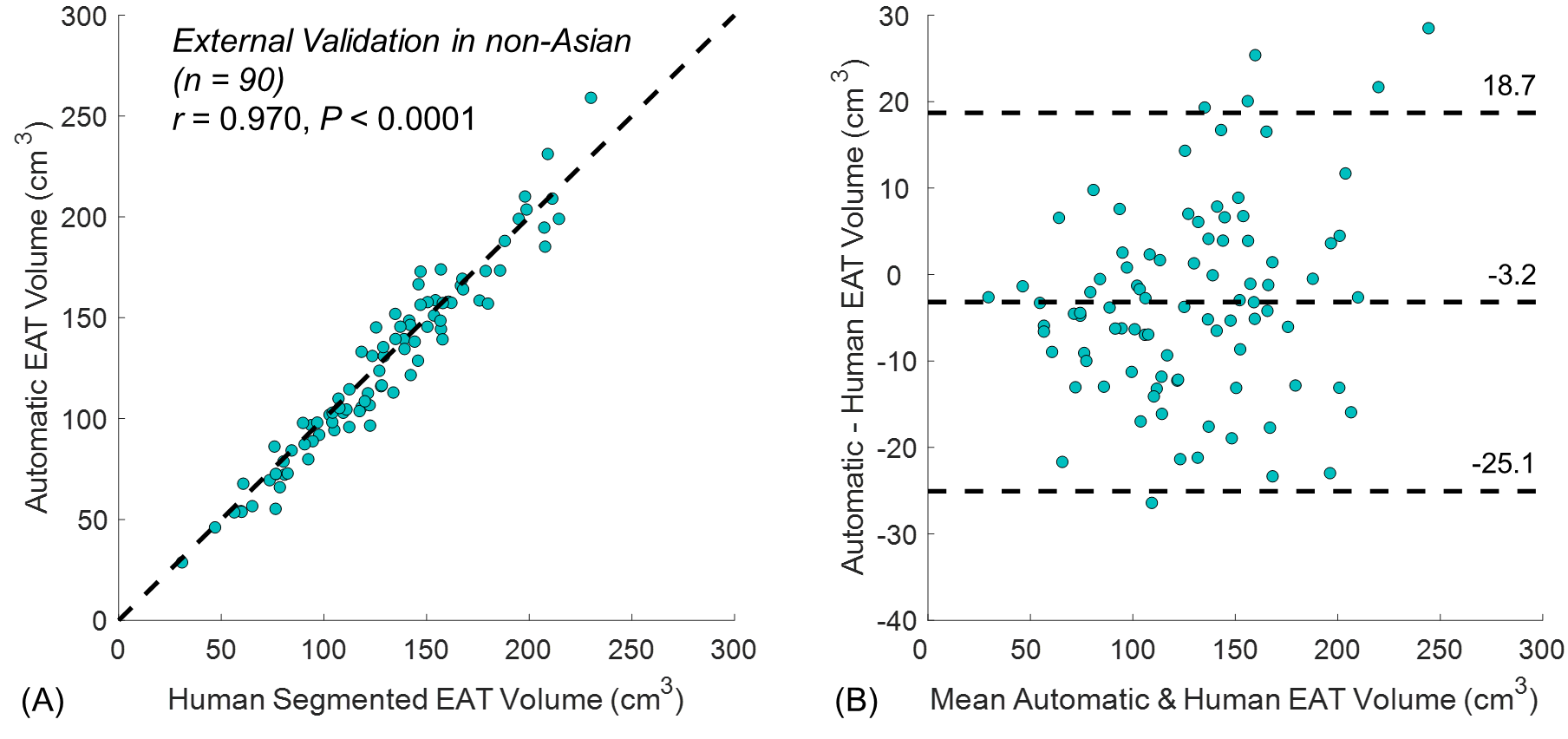


**Supplementary Figure 7.** External validation in 90 non-Asian cases. (A) Correlation plot and (B) Bland-Altman plot comparing automated and expert manual epicardial adipose tissue (EAT) volume measurements.


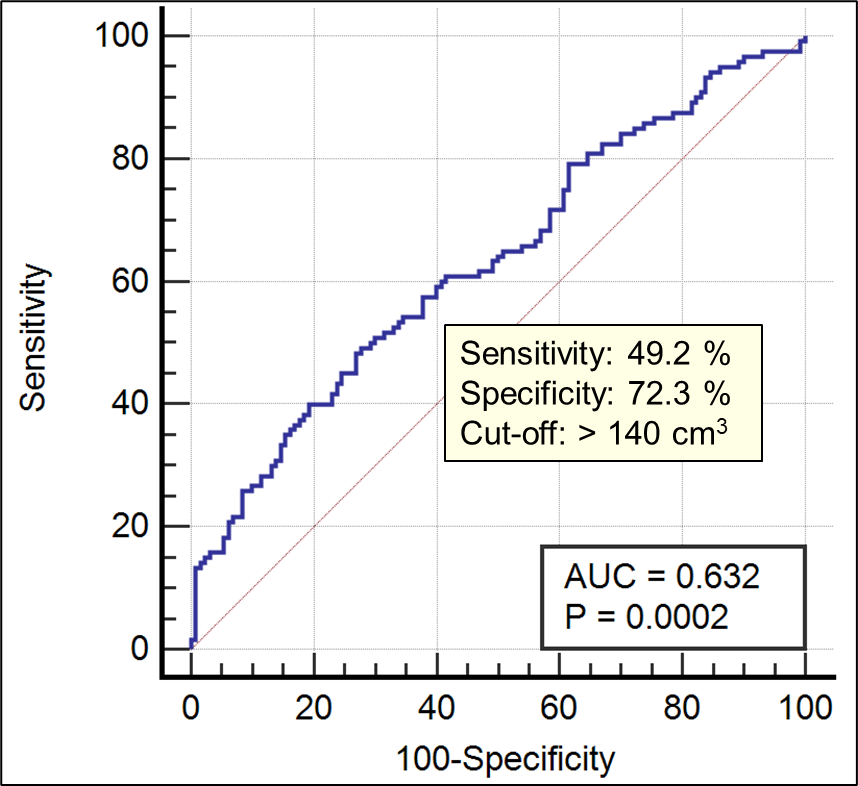


**Supplementary Figure 8.** Receiver operating characteristic (ROC) curve analysis of epicardial adipose tissue (EAT) volume for predicting obstructive coronary artery disease (CAD).
